# Glutamine Transporter SLC1A5 Regulates Ionizing Radiation-Derived Oxidative Damage and Ferroptosis

**DOI:** 10.1155/2022/3403009

**Published:** 2022-10-10

**Authors:** Zhuhui Yuan, Tong Liu, Xiao Huo, Hao Wang, Junjie Wang, Lixiang Xue

**Affiliations:** ^1^Department of Radiation Oncology, Peking University Third Hospital Cancer Center, Peking University Third Hospital, Haidian District, 49 Huayuan North Road, Beijing, China; ^2^Center of Basic Medical Research, Institute of Medical Innovation and Research, Peking University Third Hospital Cancer Center, Peking University Third Hospital, Haidian District, 49 Huayuan North Road, Beijing 100191, China; ^3^Biobank, Peking University Third Hospital Cancer Center, Peking University Third Hospital, Haidian District, 49 Huayuan North Road, Beijing 100191, China

## Abstract

Ionizing radiation-derived oxidative stress and ferroptosis are one of the most important biological effects on destroying the liver tumor, whereas radioresistance of liver tumor remains a leading cause of radiotherapy (RT) failure mainly because of the protective antiferroptosis, in which oxidative stress and subsequent lipid peroxidation are the key initiators. Thus, it is of great importance to overcome ferroptosis resistance to improve the therapeutic efficacy of RT in liver tumor patients. Irradiation-resistant HepG2 cells (HepG2-IRR) were established by long-term exposure to X-ray (2 to 8 Gy), and targeted metabolomics analysis revealed an obvious increase in intracellular amino acids in HepG2-IRR cells upon ferroptosis stress. Among these amino acids with obvious changes, N-acetylglutamine, a derivative of glutamine, is essential for the redox homeostasis and progression of tumor cells. Interestingly, the treatment of glutamine starvation could promote the ferroptosis effect significantly, whereas glutamine supplementation reversed the ferroptosis effect completely. Consistent with the changes in amino acids pattern, the glutamine transporter SLC1A5 was verified in liver tumor samples from TCGA training and validation cohorts as an independent prognostic amino acid-ferroptosis gene (AFG). A risk score for screening prognosis based on the SLC1A5, SLC7A11, ASNS, and TXNRD1 demonstrated that a high-risk score was correlated with poor survival. *In vitro* studies had shown that the knockdown of SLC1A5 resulted in a significant decrease in cell viability and promoted lipid peroxidation and oxidative damage introduced by irradiation (10 Gy). Collectively, our findings indicated that SLC1A5 may act as a suppressor gene against ferroptosis and can be a potential target for ionizing radiation mediated effects.

## 1. Introduction

Oxidative stress is one of the most important mechanisms of ionization-induced cell damage, and ferroptosis, which is triggered by oxidative stress, has been identified as a crucial mechanism involved in tumor progression and radiosensitivity [1–3]. Recently, it has been found that ferroptosis can improve radiosensitivity *in vivo* and *in vitro* [4–8]. Specifically, the type of amino acid transporters expressed in tumor cells has been widely explored in regulating ferroptosis-related radiosensitivity [4–6, 9, 10]. The Xc-transport system that carries cysteine to synthesize glutathione (GSH) for controlling intracellular redox homeostasis and defending against ferroptosis [11] has been considered an important molecular mechanism of radioresistance. The regulation of glutamine decomposition may have an impact on the susceptibility of cells to ferroptosis. The most favorable evidence is that glutamine synthase 2 (GLS2) boosts ferroptosis by facilitating the conversion of glutamate to *α*-ketoglutarate [12]. In addition, reductive glutamine metabolism is stimulated by the mTOR/PGC-1/SIRT3 pathway, one of the key signals in carcinogenesis, to decrease the oxidative stress [13]. Aberrant glutamine metabolism can act as a biological target for the radiosensitization of tumors [14]. Thus, the mechanisms of intracellular oxidative stress management upon ferroptosis activation tend to be important issues affecting radiosensitivity. The effects of amino acid metabolism implicated in redox and ferroptosis on radiosensitization require to be paid more attention.

In liver tumor, metabolic reprogramming results in tumor aggressiveness, rapid progression, and radioresistance. Amino acid metabolism and redox homeostasis, which support the requirements of exponential growth and proliferation, are critical for metabolic reprogramming [15]. According to recent studies, amino acid-derived metabolites were oncogenic in the liver. Glutamine is the predominant ingredient for nucleotide synthesis and protein synthesis, both of which are necessary for tumor cell proliferation and energy supply. Moreover, it is reported that glutamine can facilitate tumor cells to defend against oxidative stress and ferroptosis [16]. Using pharmacological approaches targeting glutamine uptake or utilization could trigger ferroptotic cell death and suppress cancer cell growth [17, 18]. However, it is still not clear which targets or pathways of amino acid metabolism are involved in ferroptosis and thereby impact irradiation response in liver tumor.

In this study, we first proved the presence of ferroptosis resistance and increased amino acid metabolism in innate or acquired radioresistant liver tumor cells. Secondly, ferroptosis was considerably promoted in liver tumor cells by glutamine starvation, whereas this effect was rescued by supplementing with glutamine. SLC1A5, as the glutamine transporter, was screened out to regulate ferroptosis response in liver tumor cell lines. Finally, through a variety of *in vitro* experiments, we identified the tumorigenic functions of SLC1A5 as well as its suppressive impact on oxidative stress-induced ferroptosis and RT. The clinical prognosis of liver tumor patients could be predicted using a risk signature based on SLC1A5-related genes. Overall, SLC1A5 may serve as a novel predictive biomarker and a possible therapeutic target for liver tumor patients who are treated with radiotherapy. Our research opens a new avenue for the treatment of radiotherapy-resistant liver tumor in patients with differential SLC1A5 expression in the future.

## 2. Methods and Materials

### 2.1. Reagents

The DMEM high-glucose culture medium was purchased from HyClone (Cat# SH30023.01), and fetal bovine serum was purchased from Gibco (Cat# 10099141). Trypsin was purchased from Invitrogen (Cat# 25200072). Erastin was obtained from Millipore (Cat# 329600). RSL3 (Cat# S8155), liproxstatin-1 (Cat# S7699), and ferrostatin-1 (Cat# S7243) were obtained from Selleck.

### 2.2. Cell Culture

Cells, including HepG2, SMMC-7721, Huh7, Huh6, and HT1080, were cultured in DMEM medium supplemented with 10% FBS, 100 IU penicillin, and 100 *μ*g/ml streptomycin and incubated at 37°C in a 5% CO_2_ humidified incubator.

### 2.3. Establishment of Radioresistant Liver Tumor Cell Line

Exponentially growing HepG2 cells were irradiated with 2, 4, 6, and 8 Gray (Gy) at a dose rate of 6 Gy/min. The radiation field was 10∗10 cm. The surviving sublines (HepG2-IRR) were then passaged at 37°C.

### 2.4. Targeted Metabolomics Analysis

Radioresistant HepG2 cells were exposed to oxidative stress by adding Erastin (50 *μ*M) for 6 hours. Cells (1.2∗10^7^) were collected. Targeted metabolomics analysis was performed using the Q300 Kit (Metabo-Profile, Shanghai, China) as described previously [19]. Briefly, ultraperformance liquid chromatography coupled to a tandem mass spectrometry (UPLC-MS/MS) system (ACQUITY UPLC-Xevo TQ-S, Waters Corp., Milford, MA, USA) was used to quantitate all targeted metabolites in this study. The details of the untargeted metabolomics analysis are listed in Supplementary file 1.

### 2.5. Lipid Peroxidation Assay

Cells (2.5∗10^5^/well) were seeded in triplicate in 6-well plates 24 hours before treatment, pretreated with or without erastin for 24 h, and then irradiated with 10 Gy at a dose rate of 6 Gy/min. Fresh media containing 0.1X BODIPY 581/591 C11 dye (Abcam, Cat# ab243377) was added to each well after the cells had been treated for 24 hours. The cells were rinsed with PBS and trypsinized to obtain a cell suspension after incubation for 20 minutes in a humidified incubator (at 37°C, 5% CO_2_). Lipid peroxidation levels were analyzed by flow cytometry using a Beckman Coulter CytoFLEX™ (CytoFLEX) and analyzed with FlowJo software.

### 2.6. Quantitative Real-Time Polymerase Chain Reaction (qRT-PCR)

Total RNA was extracted according to the manufacturer's instructions using TRIzol® reagent (Invitrogen, Cat# 15596018). The HiScript III 1st Strand cDNA Synthesis Kit (Vazyme, Cat# R312-01) was used to reverse transcribe 1 g total RNA into single-strand complementary DNA (cDNA). Following the manufacturer's procedures, RT-qPCR was done in triplicate using SYBR Green PCR Master Mix. GAPDH was used as a reference to standardize the relative expression levels of SLC1A5. The 2^-*ΔΔ*Ct^ method was used to calculate gene expression. The sequences of the primers used for RT-qPCR were listed as follows:

1. SLC1A5 forward: GTGTCCTCACTCTGGCCATC
2. SLC1A5 reverse: CCCAGAGCGTCACCTTCTAC

### 2.7. CCK8 Assay

Cells (1∗10^4^/well) were seeded into 96-well plates. To determine the effect of treatment on cell viability, a 10% volume of CCK8 (Dojindo Laboratories, Cat# CK04) was added directly into the medium and incubated for 3 hours. Absorbance at wavelengths of 450 nm was measured. Results were normalized to untreated controls and shown as relative cell viability (%).

### 2.8. Data and Resources

The RNA-Seq data and clinical information for the liver hepatocellular carcinoma (LIHC) project were derived from TCGA (https://portal.gdc.cancer.gov/, updated time of datasets: 20200814). We downloaded RNA-Seq data expressed as fragments per kilobase of exon per million reads mapped (FPKM) from TCGA database. Ensembl IDs were transformed into gene symbols. The median value was used as the gene symbol's expression profile when more than one ensemble ID matched the same gene symbol. The mRNA data of TCGA dataset contains 374 tumor samples and 50 normal samples. Tumor samples with missing clinical data or follow-up periods < 6 months were censored, and the remaining tumor sample in TCGA dataset was 334.  Table 1 displays the clinical features of the liver tumor patients in TCGA. For gene expression analysis, All TCGA liver tumor patients were randomly divided into training cohort (number of patients: 168) and validation cohort (number of patients: 166). The patients in the training and validation cohort were further divided into the high- and low-risk groups based on the median value of the risk score. The RNA-Seq data of GSE94550 and GSE123062 were derived from GEO datasets (https://www.ncbi.nlm.nih.gov/geo/) and analyzed with GEO2R (https://www.ncbi.nlm.nih.gov/geo/geo2r/, adjust *p* value less than 0.05 were considered differentially expressed).

### 2.9. Cox Regression Analysis

Cox regression analysis was performed with the “survival” package in R using TCGA datasets. The AFGs that differed in expression between tumor tissue and its surrounding normal tissues and had *p* values less than 0.05 were classified as prognostic related-amino acid-ferroptosis genes (AFGs).

### 2.10. Statistical Analysis

Statistical analysis was performed using R (version 3.6.3, http://www.r-project.org/) and GraphPad Prism 8.0 (GraphPad Prism Software Inc., San Diego, CA, USA). Unpaired Student's *t*-tests or one-way analyses of variance with a Bonferroni post hoc test for multiple group comparisons were used to identify significant differences between the groups. *p* < 0.05 was a statistically significant difference. For mRNA seq data from TCGA, genes with |logFC| ≥ 1 and FDR < 0.05 (FC means fold change; FDR means false discovery rate) were considered significantly differentially expressed.

## 3. Results

### 3.1. The Content of Amino Acids Is Increased in Radioresistant Liver Tumor Cells upon Oxidative Stress

**Figure 1:**
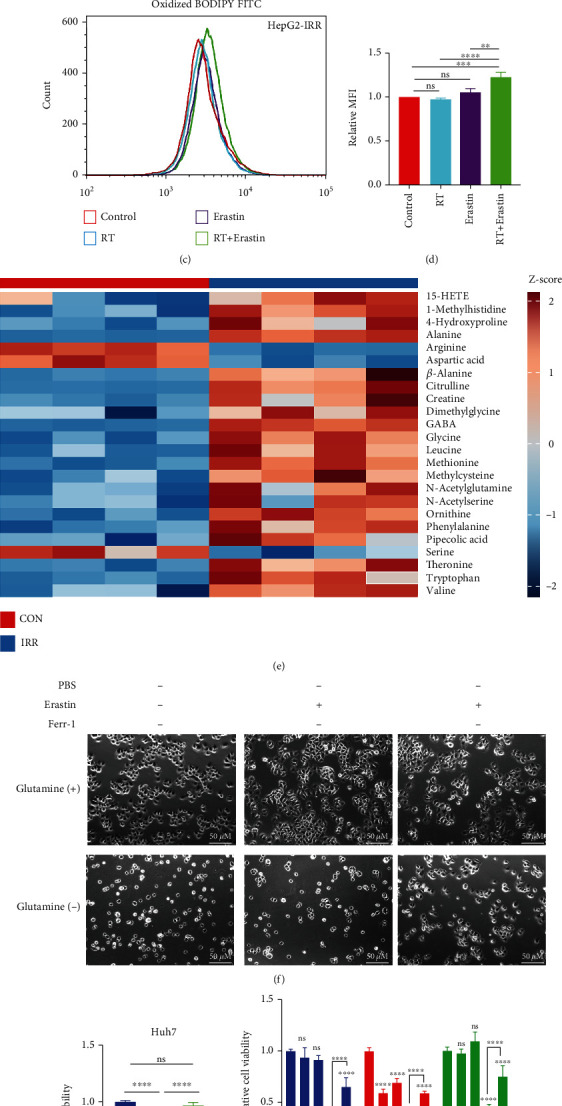
A combination of RT and ferroptosis inducer promotes ferroptotic cell death in the liver tumor cell lines. (a, b) Flow cytometry revealed a significant increase in normalized MFI of lipid peroxidation in SMMC-7721 cells treated with RT (8 Gy) after pretreatment with erastin (25 *μ*M). (c, d) In HepG2-IRR cells, RT (8 Gy) in combination with Erastin (25 *μ*M) enhances the normalized MFI of lipid peroxidation as determined by flow cytometry. (e) After Erastin (25 *μ*M) stimulation, the hierarchical clustering of targeted metabolic comparing HepG2-IRR cells and wild-type HepG2 cells indicates a noticeable increase in amino acid content. (f) Glutamine is essential for SMMC-7721 cell survival, and glutamine starvation induces ferroptosis in SMMC-7721 cells which can be reversed by ferr-1. Erastin: 25 *μ*M; Ferr-1: 1 *μ*M. Magnification: 20X; scale bar: 50 *μ*M. (g, h) The sensitized ferroptosis effect of glutamine deprivation is reversed by glutamine supplementation. Huh7, Huh6, HepG2, and HT1080 cell lines are more susceptible to ferroptotic cell death after being treated with RSL3 (2.5 *μ*M) and glutamine deprivation for 24 hours, and glutamine supplementation can entirely reverse this effect. Abbreviations: RT: radiotherapy; IRR: irradiation-resistant; MFI: mean fluorescence intensity; AFGs: amino acid-ferroptosis genes. ^∗^*p* < 0.05;  ^∗∗^*p* < 0.01;  ^∗∗∗^*p* < 0.001;  ^∗∗∗∗^*p* < 0.0001.

Since irradiation can give rise to ferroptosis to eliminate tumor cells, we first sought to detect if the innate or acquired resistance of liver tumor cells to irradiation could attenuate ferroptosis stress. HepG2 and SMMC-7721 cell lines are commonly utilized liver tumor cell lines in radiobiological and ferroptosis studies [20, 21]. As shown in Figures 1(a)–1(d), RT or Erastin (one of the commonly used ferroptosis inducers) alone increased the mean fluorescence intensity (MFI) of lipid peroxidation (LPO) slightly, a standard marker of ferroptosis activation detected by BODIPY581/591 probe [22], whereas the combination of RT and Erastin increased MFI significantly in SMMC-7721 cells and radioresistant HepG2 cells (HepG2-IRR) which we had reported previously [23]. These findings suggested that liver tumor cells, whether they have intrinsic or acquired radioresistance, display limited sensitivity to ferroptosis stress; however, liver tumor cells' response to ferroptosis can be improved by amplifying ferroptosis stress, such as utilizing a combination method. Therefore, to improve the clinical outcome of liver tumor patients receiving RT, it is essential to explore the critical factor(s) that have an impact on ferroptosis response.

The transportation and *in vivo* utilization of amino acids play a significant role in ferroptosis and RT response as known. To verify whether amino acid metabolism alterations have an impact on ferroptosis and RT response in liver tumor cells, we performed targeted metabolomics to evaluate significantly altered metabolites. Upon treatment of Erastin, targeted metabolomics revealed that the content of amino acids (AA) differed significantly in HepG2-IRR cells (FDR-corrected *p* < 0.05; FC > 1.5) compared to wild-type HepG2 cells (Supplementary Figure S1), especially N-acetylglutamine, glycine, leucine, methionine, threonine, tryptophan, and valine (Figure 1(e)). N-acetylglutamine is a derivative of glutamine, and glutamine is essential for the proliferation and development of tumor cells. Consistent with this, SMMC-7721 cells survived in the absence of glutamine either in the ferroptosis condition or the basal condition (Figure 1(f)), and this effect was significantly reversed by a ferroptosis-specific inhibitor (Ferr-1). Additionally, compared to another commonly used ferroptosis inducer—RSL3, glutamine deprivation combined with RSL3 significantly promoted ferroptotic cell death, and glutamine supplementation significantly reversed ferroptotic cell death triggered by the combination of glutamine deprivation and RSL3 (Figures 1(g) and 1(h)). These findings strongly indicated that glutamine is required for tumor cell proliferation and that glutamine deficiency can lead to ferroptotic cell death [24].

### 3.2. Glutamine Membrane Transporter SLC1A5 Is Upregulated in Liver Tumor and Indicates a Poor Prognosis

To validate the effect of glutamine on ferroptosis and explore the intrinsic origins, we next downloaded the transcriptome data of liver tumor and the corresponding clinical data from TCGA database to detect the potential regulated gene for amino acid metabolism-mediated ferroptosis response.

**Figure 2:**
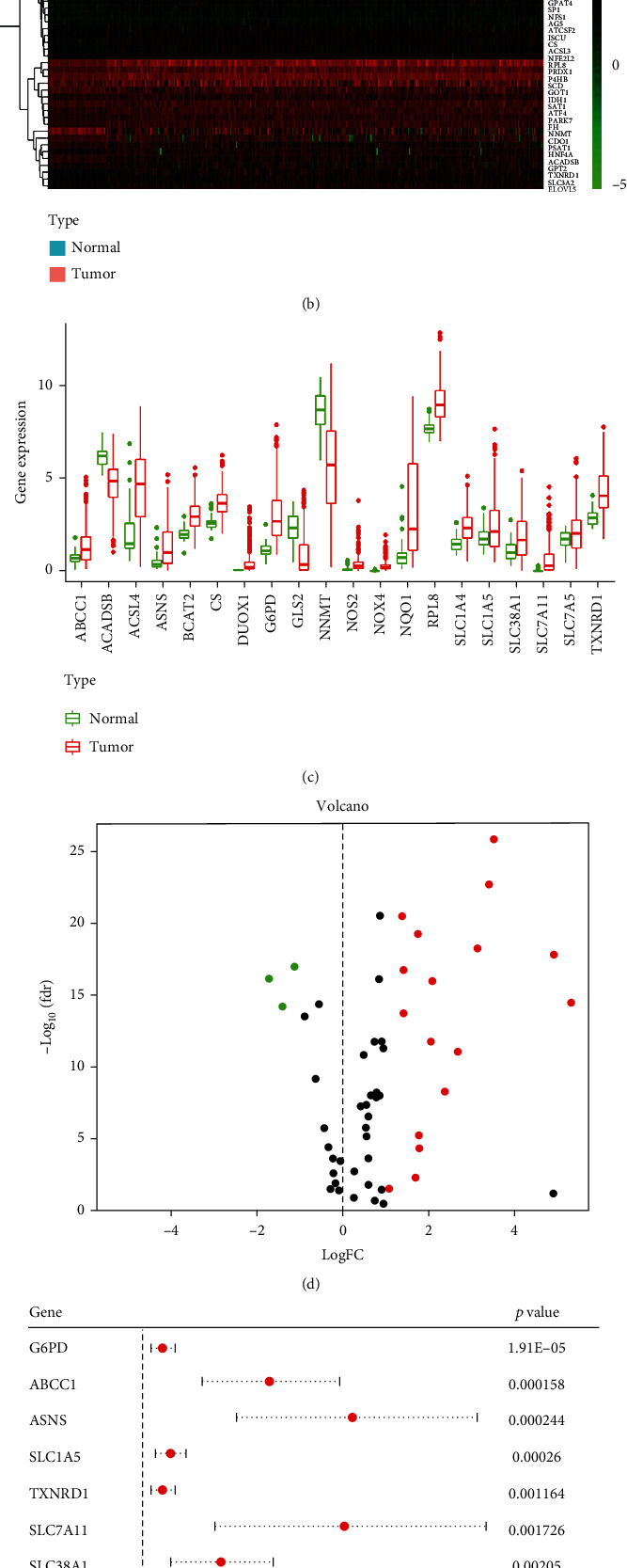
The expression of thirty-four intersecting genes in the tumor and surrounding normal tissues of the liver. (a) Venn diagram indicates 54 intersecting genes (AFGs) involved in both ferroptosis and amino acid metabolism. (b) Hierarchical clustering of the 54 intersecting genes in liver tumor tissue and normal tissue from TCGA database. (c) The expression difference of the 54 intersecting genes between liver tumor tissue and normal tissues is depicted by a volcano heat map. A total of 20 intersecting genes shows a significant difference. The intersecting genes with increasing levels in tumor tissues are shown in the right panel (red), whereas the intersecting genes with lower levels in tumor tissues are shown in the left panel (green). (d) The expression of the 20 AFGs in tumor and normal tissues of the liver. (e) The findings of the univariate Cox regression analysis between 20 AFG expressions and OS is shown in forest plots. Nine genes correlated with OS are detected. There are 3 downregulated DEGs (NNMT, GLS2, and ACADSB) and 17 upregulated DEGs (SLC1A5, SLC1A4, SLC7A11, etc.). (f) Multivariate Cox analysis shows eight AFGs negatively correlated with OS in liver tumor. AFGs: amino acid-ferroptosis genes.

Firstly, to obtain the list of the genes involved in both amino acid metabolism and ferroptosis, we merged the ferroptosis genes (Supplementary file 2) from FerrDb databases and amino acid metabolism-related genes (Supplementary file 3) from the GSEA datasets. The Venn diagram suggested 54 genes associated with amino acid metabolism related-ferroptosis genes (AFGs) (Figure 2(a)). The expression of the 54 genes between liver tumor tissues and surrounding normal tissues in TCGA dataset is shown in Figure 2(b). According to the threshold of logFC (|logFC| ≥ 1) and FDR (FDR < 0.05), 20 AGFs were expressed differentially (DEGs, Figures 2(c) and 2(d) and Supplementary file 4): there were 3 downregulated DEGs (NNMT, GLS2, and ACADSB) and 17 upregulated DEGs (SLC1A5, SLC1A4, SLC7A11, etc.).

**Figure 3:**
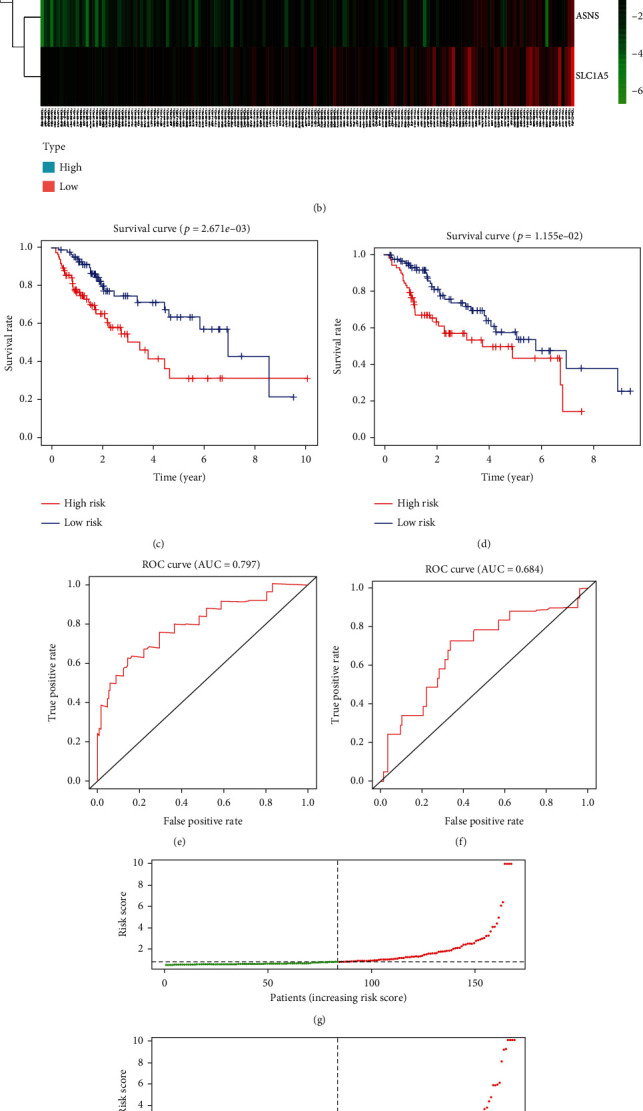
liver tumor patients with a high-risk score suggest a lower RT response and a higher survival risk, compared with liver tumor patients with a low-risk score. (a, b) According to the results of cox regression, 4 AFGs are screened to establish a prognostic model. The 4 AFGs are all highly expressed in liver tumor tissues. (c, d) Kaplan-Meier survival curves comparing liver tumor patients' OS based on the expression of the five AFGs indicate that the five AFGs are negatively associated with OS in liver tumor. The Kaplan-Meier graphs demonstrate the difference in OS between the high-risk and low-risk groups in the training cohort (a) and validation cohort (b). Patients in the high-risk group show poor survival compared with low-risk group patients. AUC of time-dependent ROC curves verifies the prognostic performance of the risk score in the training cohort (e) and validation cohort (f). The distribution and median value of the risk scores in the training cohort (g) and validation cohort (h). The distributions of OS status, OS, and risk score in the training cohort (i) and validation cohort (j). AFGs: amino acid-ferroptosis genes.

Next, we evaluated the correlation between AFGs and overall survival (OS) data from TCGA dataset. COX regression analysis showed 9 AFGs with *p* < 0.05 (Figure 2(e)) as the potential prognostic AFGs. Moreover, multivariate Cox regression analysis was performed to explore the independent prognostic genes (Figure 2(f), TXNRD1, ASNS, SLC7A11, and SLC1A5) and construct a four-gene risk signature model (Figures 3(a)–3(j), and Supplementary file 5).

**Figure 4:**
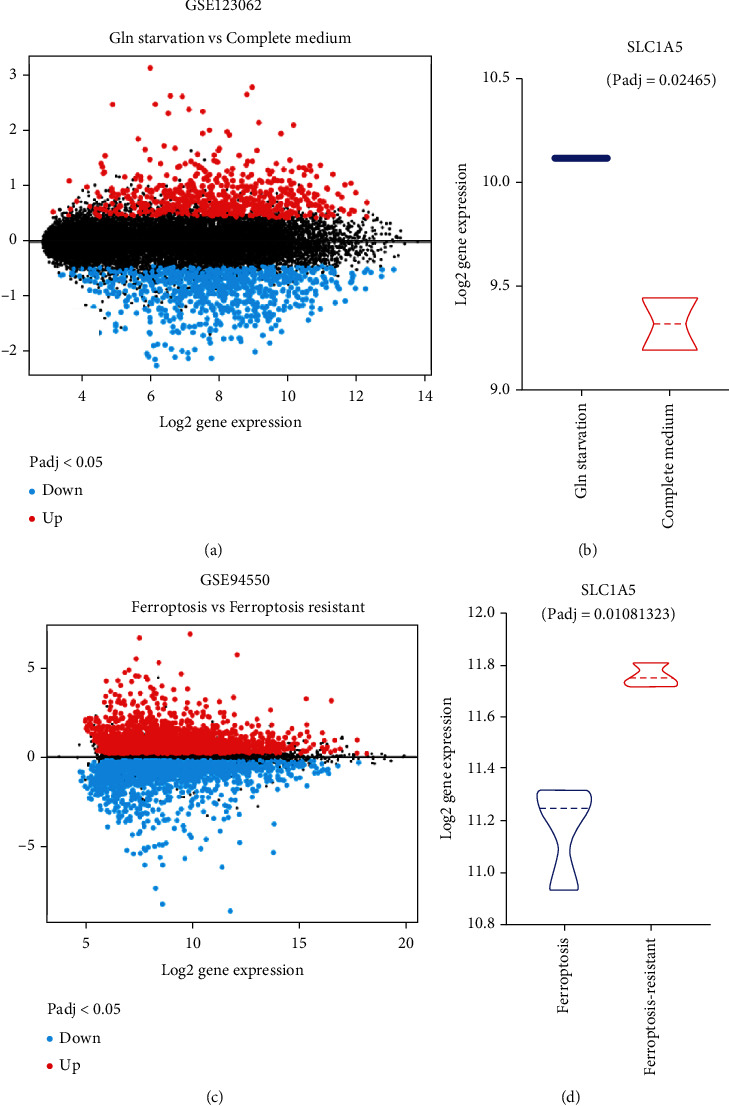
SLC1A5 is associated with glutamine starvation and ferroptosis response in the liver tumor. (a, b) SLC1A5 reactively increases when liver tumor cells suffer glutamine starvation (GSE123062). The volcano map displays the differently expressed genes between liver tumor cells treated with glutamine and those treated without glutamine (a). SLC1A5 is upregulated during glutamine starvation (b). (c, d) Huh7 cells with response to ferroptosis inducer sorafenib have lower expression level of SLC1A5, compared to those without response to ferroptosis.

Among the four independent prognostic genes, SLC1A5 is the critical transporter for glutamine uptake and a suppressor gene in ferroptosis. In liver tumor tissue, the expression of SLC1A5 was significantly higher than in normal liver tissues (Figures 2(b) and 2(c)). As shown in Figures 4(a) and 4(b), SLC1A5 reactively increased when liver tumor cells suffered glutamine starvation. Moreover, Huh7 cells with response to ferroptosis inducer sorafenib had lower expression level of SLC1A5, compared to those without response to ferroptosis (*p* = 0.01081323, Figures 4(c) and 4(d)). Collectively, these findings suggested that SLC1A5 is upregulated in liver tumors, and the expression level of SLC1A5 is correlated with liver tumor patients' prognosis.

Gene risk signature reveals that high expression of SLC1A5 is tightly related to a high risk of death.

To further evaluate the predictive power of SLC1A5 in prognostics of liver tumor patients, all TCGA liver tumor patients were randomly divided into training cohort (*n* = 168) and validation cohort (*n* = 166). The patients in the training and validation cohort were further divided into the high- and low-risk groups based on the median value of the risk score. In the training cohort, there were 84 patients in each of the high- and low-risk groups. In the validation cohort, 73 patients were in the high-risk group and 93 patients were in the low-risk group. The risk scores of all liver tumor patients were calculated as follows: TXNRD1∗0.011594283 + ASNS∗0.11892719 + SLC1A5∗0.014906744 + SLC7A11∗0.122043117. The expression of the four AFGs in training and validation cohorts is shown in Figures 3(a) and 3(b). The Kaplan-Meier (KM) survival curves demonstrated that the prognosis of liver tumor patients in the low-risk group was significantly higher than that in the high-risk group in both the train and validation cohorts (Figures 3(c) and 3(d)). In the training and validation cohorts, the areas under the curves (AUCs) of the time-dependent receiver operating characteristic (ROC) curves of the predicted 1-year risk OS were 0.797 and 0.684, respectively (Figures 3(e) and 3(f)). Between the two groups, there were significant differences in the survival curve and status (Figures 3(g) and 3(h)). The death cases in the high-risk group are significantly higher than those in the low-risk group.

### 3.3. Loss of SLC1A5 Is Involved in Radiosensitization

**Figure 5:**
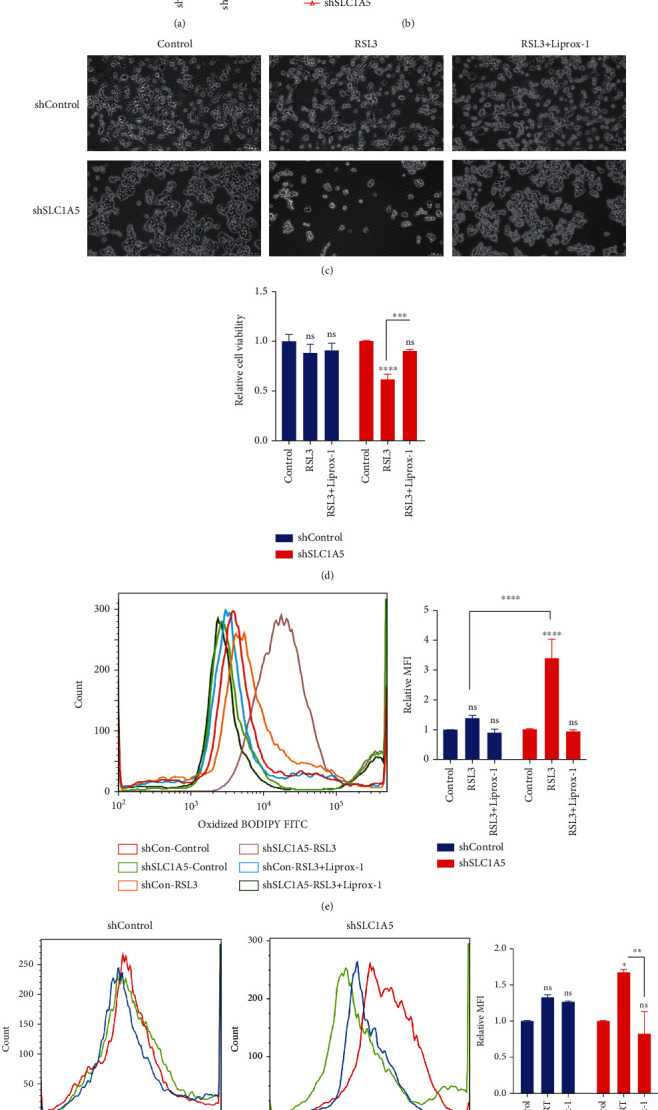
Glutamine transporter SLC1A5 affected ferroptosis response in liver tumor cell lines. (a) The knockdown efficiency of SLC1A5 in Huh7 cells was determined by qPCR. (b) In shSLC1A5 cells, the results of CCK8 demonstrated a significant reduction in tumor cell proliferation. (c) After RSL3 stimulation (2.5 *μ*M for 6 hours), the level of cell viability in shSLC1A5 Huh7 cells was much lower than it was in shControl cells. (d) In shSLC1A5 cells, the MFI of LPO increased substantially, and the elevated MFI of LPO was reversed by a ferroptosis inhibitor. (e) After Erastin stimulation (25 *μ*M for 24 hours), the level of cell viability in shSLC1A5 Huh7 cells was much lower than it was in shControl cells. Lirpoxstatin-1 could reverse the enhanced ferroptosis effect induced by knock-down of SLC1A5. (f) shSLC1A5 dramatically improved the liver tumor cells' sensitivity to irradiation-induced LPO accumulation. RT: 8 Gy. AFGs: amino acid-ferroptosis genes; ferr-1: ferrostatin-1. ^∗^*p* < 0.05;  ^∗∗^*p* < 0.01;  ^∗∗∗^*p* < 0.001;  ^∗∗∗∗^*p* < 0.0001.

To further confirm the function of SLC1A5 at a cellular level, we utilized lentivirus transduction to knock down SLC1A5 using an shRNA vector that specifically targets SLC1A5 (shSLC1A5) in Huh7 cells. As shown in Figure 5(a), shSLC1A5 resulted in a 70% reduction of SLC1A5 mRNA when compared to a control shRNA vector in Huh7 cells. In shSLC1A5 cells, the results of CCK8 demonstrated a significant reduction in tumor cell proliferation (Figure 5(b)). The results showed that SLC1A5 deletion abolished Huh7 cell growth *in vitro*.

Next, we explored the effect of SLC1A5 on ferroptosis and radiosensitivity. After ferroptosis stimulation, the level of cell viability in SLC1A5 null Huh7 cells was much lower than it was in shRNA control cells (Figures 5(c) and 5(e)). In SLC1A5 null cells, the MFI of LPO increased substantially, and the elevated MFI of LPO was reversed by a ferroptosis inhibitor (Figure 5(d)). Moreover, the absence of SLC1A5 dramatically improved the liver tumor cells' sensitivity to irradiation-induced LPO accumulation (Figure 5(f)). Collectively, these results indicated that SLC1A5 was a ferroptosis suppressor gene in liver tumor cells, and deletion of SLC1A5 may boost RT response by enhancing the ferroptosis effect.

## 4. Discussion

Recently, the effects of ferroptosis in radiotherapy have attracted much interest. Previous investigations highlighted the fatal consequences of lipid peroxides introduced by ferroptosis and irradiation, as well as the synergistic effect of ferroptosis on radiotherapy [4, 6, 25]. According to some research, amino acids may influence tumor response to irradiation via modulating ferroptosis signals [6, 26, 27]. However, ferroptosis-dependent radiosensitivity is a complicated issue, and the mechanism governing amino acid metabolism in ferroptosis remains obscure. We verified that amino acids played a significant role in ferroptosis-regulated radiosensitivity and identified 4 independently prognostic AFGs (SLC1A5, SLC7A11, ASNS, and TXNRD1) as prospective candidates for predicting ferroptosis and irradiation response. Amino acid transportation and metabolism regulate ferroptosis and provide new opportunities for developing radiation-resistant targeted therapies for liver tumor.

In the current study, we discovered that liver tumor cells resisted irradiation by increasing their amino acid content to defend against ferroptosis. These findings were consistent with previous investigations. Patients with nonalcoholic steatohepatitis (NASH), one of the precancerous diseases of liver tumor, showed a high concentration of glutamate in circulation [28]. Increased glutamine was primarily used to generate GSH to overcome oxidative stress, particularly in liver tumor patients with a history of NASH. Since both RT and ferroptosis have a fatal effect on tumor cells by increasing oxidative stress, a high number of intracellular antioxidants can prevent ferroptotic cell death and promote liver tumor progression. Our findings not only investigated the metabolic characteristics of radioresistance liver tumor cells upon ferroptosis stress but also demonstrated that glutamine was required for liver tumor cells' survival. Glutamine deprivation induced ferroptosis in liver tumor cells, which would be reversed by ferroptosis inhibitors, indicating that targeting glutamine uptake could be a therapeutic strategy for liver tumor treatment. These findings could pave the way for a new approach to adjuvant therapy that targets or alters amino acid metabolism to improve radiation response in liver tumor. Focusing on the impact of glutamine starvation on ferroptosis, however, should take into account the number and kind of deficient amino acids, time window, and heterogeneity of tumors or tumor cell lines. Based on the presence of transferrin and the stress of multiple amino acid deprivation, Gao et al.'s work showed that glutamine was required for inducing ferroptosis in MEF cells [29]. It should be noted that Gao et al.'s study uses a design of multiple amino acid deprivation rather than a single amino acid deprivation and that the time window is 12 hours, indicating an early response to nutrient stress. In contrast, we just deplete glutamine and assess the tumor cells' response 24 hours later, which may reflect an intermediate or advanced response to nutrient stress. Therefore, distinct phenotypes in tumor cells may be induced depending on the type of deficient amino acids and the time window. Additionally, the heterogeneity of various tumors or diverse tumor cell lines may also play a significant role in how cancers respond to amino acid deprivation. For example, the sensitivity of a panel of lung cancer cell lines to glutamine deprivation varied significantly, with some cells exhibiting almost total independence [30]. Additionally, basal-type cells tend to be glutamine-dependent, whereas luminal-type cells tend to be glutamine-independent in breast cancer cells, demonstrating systematic distinctions in glutamine dependence [31]. However, in the current study, the HT1080 cell line and diverse liver tumor cell lines (HepG2, Huh7, and SMMC-7721) show vulnerability to ferroptosis caused by glutamine starvation. Similar results were also reported in Wappler et al.'s [32] investigation: chemoresistance was entirely reversed in glutamine-depleted cholangiocarcinoma. Qing et al. [33] also demonstrated that glutamine deprivation induces cell death in neuroblastoma. Therefore, more research is needed to better determine how glutamine deprivation affects ferroptosis in various tumor types. SLC1A5 is a critical glutamine transporter involved in ferroptosis. In the current study, we found that knockdown of SLC1A5 increased liver tumor cells' response to irradiation-induced oxidative damage at a dose of 10 Gy. SLC1A5 has been regarded as an oncogene by the mTORC1 signaling pathways or KRAS mutation in certain tumors [34, 35], and loss of SLC1A5 may inhibit tumor growth. Furthermore, targeting SLC1A5 has been shown to sensitize ferroptosis in melanoma [18]. In liver tumor, some studies demonstrated the role of SLC1A5 as a risk factor [36–38], which were consistent with our findings. These findings indicate that targeting SLC1A5 tends to be an effective approach to improve liver tumor patients' prognosis. However, it is worth noting that SLC1A5 is not the only glutamine transporter. Indeed, SLC38A1 has been identified as a rescue transporter for compensating glutamine uptake [39, 40]. SLC1A5 and SLC38A1 cotransport polarized Na^+^ and glutamine, and SLC1A5 has been identified as amino acid harmonizer, whereas SLC38A1 has been recognized as an amino acid loader in cancer cells [40]. However, the roles of SLC1A5 and SLC38A1 in different tumor types are still under debate. However, in some tumor cell lines, such as 143B osteosarcoma cells, loss of SLC1A5 did not suppress tumor growth, but instead elicit an amino acid starvation response and up-regulation of SLC38A1, indicating that SLC38A1 may act as a rescue transporter when SLC1A5 is blocked [40]. Therefore, the functional complementarity between SLC1A5 and SLC38A1 is intertwined, and targeting both SLC1A5 and SLC38A1 could be further evaluated for their tumor suppression capability. Besides, when evaluating the impact of SLC1A5 and SLC38A1 on ferroptosis, different tumor types and heterogeneity should also be carefully considered.

We also found a SLC1A5-based risk signature for predicting prognosis. In ferroptosis, SLC7A11 is one of the most important suppressor genes [6, 41, 42]. SLC7A11 and SLC3A2 compose the cystine antiporter system Xc-. Cystine is utilized for GSH synthesis [43]. Irradiation has been shown to suppress SLC7A11 protein expression by activating TP53 and inducing ferroptosis cell death [26]. Moreover, ferroptosis inducers inhibited SLC7A11 in a synergistic effect with RT [6]. In addition, tumors with a high level of SLC7A11 showed endogenous resistance to ferroptosis and RT. Within our four-factor risk-predicting model, TXNRD1 is a selenocysteine-containing flavoenzyme, and the enzyme activity is mainly affected by intracellular ROS [44]. Though TXNRD1 was increased in response to cysteine starvation [45], whether TXNRD1 would affect amino acid transportation or synthesis remains unclear. The research about the role of TXNRD1 in amino acid transportation or synthesis is worthy of further studies.

There are some limitations to this study. First, prospective studies should be conducted to confirm these findings, especially the predictive capability of the proposed model. Secondly, the suppressive effect of SLC1A5 on ferroptosis should be explored in further studies, particularly considering the interaction of SLC1A5 and SLC38A1. Besides that, whether the tumor microenvironment responded similarly or differently to SLC1A5 inhibition should be validated *in vivo* and *in vitro.* Next, the role of SLC1A5 on the biological effect of high-dose and low-dose irradiation should be elucidated in further studies.

## 5. Conclusion

Taken together, changes in amino acid metabolism hints that amino acid transportation may be crucial indicators. Given the importance of glutamine for liver tumor cells, efforts toward the qualification of biomarkers for liver tumor in clinical practice are urgent. Moreover, pharmaceutical techniques to induce ferroptosis and boost radiation response could be developed by targeting amino acid metabolism, such as transportation mediated by SLC1A5.

## Figures and Tables

**Table 1:** The clinical characteristics of liver tumor patients from TCGA dataset.

Characteristics	Training (*n* = 168)	Validation (*n* = 166)	Total (*n* = 334)	*p* value
Age				
Mean ± SD	59.45 ± 12.98	58.98 ± 13.69	59.21 ± 13.32	
Gender				1.00
FEMALE	53 (15.87%)	52 (15.57%)	105 (31.44%)	
MALE	115 (34.43%)	114 (34.13%)	229 (68.56%)	
Grade				0.94
G1	27 (8.08%)	25 (7.49%)	52 (15.57%)	
G2	80 (23.95%)	76 (22.75%)	156 (46.71%)	
G3	53 (15.87%)	56 (16.77%)	109 (32.63%)	
G4	5 (1.50%)	7 (2.10%)	12 (3.59%)	
Unknown	3 (0.90%)	2 (0.60%)	5 (1.50%)	
Stage				0.46
Stage I	80 (23.95%)	78 (23.35%)	158 (47.31%)	
Stage II	31 (9.28%)	43 (12.87%)	74 (22.16%)	
Stage III	3 (0.90%)	0 (0.0e+0%)	3 (0.90%)	
Stage IIIA	31 (9.28%)	28 (8.38%)	59 (17.66%)	
Stage IIIB	3 (0.90%)	4 (1.20%)	7 (2.10%)	
Stage IIIC	6 (1.80%)	3 (0.90%)	9 (2.69%)	
Stage IV	1 (0.30%)	0 (0.0e+0%)	1 (0.30%)	
Stage IVB	1 (0.30%)	1 (0.30%)	2 (0.60%)	
Unknown	12 (3.59%)	9 (2.69%)	21 (6.29%)	
T				0.28
T1	86 (25.75%)	79 (23.65%)	165 (49.40%)	
T2	32 (9.58%)	47 (14.07%)	79 (23.65%)	
T2a	1 (0.30%)	0 (0.0e+0%)	1 (0.30%)	
T2b	0 (0.0e+0%)	1 (0.30%)	1 (0.30%)	
T3	23 (6.89%)	20 (5.99%)	43 (12.87%)	
T3a	16 (4.79%)	8 (2.40%)	24 (7.19%)	
T3b	1 (0.30%)	4 (1.20%)	5 (1.50%)	
T4	7 (2.10%)	6 (1.80%)	13 (3.89%)	
TX	1 (0.30%)	0 (0.0e+0%)	1 (0.30%)	
Unknown	1 (0.30%)	1 (0.30%)	2 (0.60%)	
M				0.83
M0	120 (35.93%)	121 (36.23%)	241 (72.16%)	
M1	2 (0.60%)	1 (0.30%)	3 (0.90%)	
MX	46 (13.77%)	44 (13.17%)	90 (26.95%)	
N				0.24
N0	115 (34.43%)	119 (35.63%)	234 (70.06%)	
N1	3 (0.90%)	0 (0)	3 (0.90%)	
NX	50 (14.97%)	46 (13.77%)	96 (28.74%)	
Unknown	0 (0)	1 (0.30%)	1 (0.30%)	

## Data Availability

The datasets analyzed during the current study are available from the corresponding author on reasonable request.

## Supplementary Materials

Supplementary Materials Figure S1: the pie graph of targeted metabolomics. HepG2 cell line and HepG2-IRR cells were treated with Erastin (50 *μ*M) for 6 hours, and cells were collected for targeted metabolomics. The content of amino acids (AA) accounting for 25% was the most abundant metabolites in HepG2-IRR cells (FDR-corrected *p* < 0.05; FC > 1.5), compared to wild-type HepG2 cells. Fatty acids account for 21% in HepG2-IRR cells after Erastin stimulation. Supplementary 1. The materials and protocol of targeted metabolomics. In brief, HepG2 cell line and HepG2-IRR cells were treated with Erastin (50 *μ*M) for 6 hours, and cells were collected for targeted metabolomics. An ultraperformance liquid chromatography coupled to tandem mass spectrometry (UPLC-MS/MS) system was used to quantitate metabolites. Supplementary 2. The list of amino acid metabolism genes. Supplementary 3. The list of ferroptosis-related genes. Supplementary 4. Twenty amino acid-ferroptosis genes were identified as prognostic factors for predicting the prognosis of liver tumor patients. Supplementary 5. SLC1A5, SLC7A11, TXNRD1, and ASNS are the independent prognostic factors for liver tumor patients.
