# Supplementary material for: Glutamine Transporter SLC1A5 Regulates Ionizing Radiation-Derived Oxidative Damage and Ferroptosis
Source: Oxid Med Cell Longev. 2022 Oct 10;2022:3403009. doi: 10.1155/2022/3403009 (PMC9576409; doi:10.1155/2022/3403009)
Supplement: Supplementary Materials — Figure S1: the pie graph of targeted metabolomics. HepG2 cell line and HepG2-IRR cells were treated with Erastin (50 μM) for 6 hours, and cells were collected for targeted metabolomics. The content of amino acids (AA) accounting for 25% was the most abundant metabolites in HepG2-IRR cells (FDR-corrected p < 0.05; FC > 1.5), compared to wild-type HepG2 cells. Fatty acids account for 21% in HepG2-IRR cells after Erastin stimulation. Supplementary 1. The materials and protocol of targeted metabolomics. In brief, HepG2 cell line and HepG2-IRR cells were treated with Erastin (50 μM) for 6 hours, and cells were collected for targeted metabolomics. An ultraperformance liquid chromatography coupled to tandem mass spectrometry (UPLC-MS/MS) system was used to quantitate metabolites. Supplementary 2. The list of amino acid metabolism genes. Supplementary 3. The list of ferroptosis-related genes. Supplementary 4. Twenty amino acid-ferroptosis genes were identified as prognostic factors for predicting the prognosis of liver tumor patients. Supplementary 5. SLC1A5, SLC7A11, TXNRD1, and ASNS are the independent prognostic factors for liver tumor patients. [file 3403009.f1.zip › supplementart 2.docx]

CSGALNACT2

CSGALNACT1

AC098591.2

TNFRSF11B

TMEM126B

SLC9A3R1

SLC7A5P2

SLC7A5P1

SLC66A1L

SLC38A11

SLC38A10

SLC30A10

SLC25A48

SLC25A47

SLC25A45

SLC25A44

SLC25A38

SLC25A32

SLC25A29

SLC25A22

SLC25A21

SLC25A20

SLC25A19

SLC25A18

SLC25A15

SLC25A13

SLC25A12

SLC25A10

SLC22A16

SLC16A12

SLC16A10

SECISBP2

RAB3GAP1

HSD17B12

HSD17B10

HS3ST3B1

GLYATL1B

ALDH18A1

AASDHPPT

TSPOAP1

TRMT10C

SULT6B1

SULT4A1

SULT2B1

SULT2A1

SULT1E1

SULT1C4

SULT1C3

SULT1C2

SULT1B1

SULT1A4

SULT1A3

SULT1A2

SULT1A1

ST3GAL6

ST3GAL4

ST3GAL3

ST3GAL2

ST3GAL1

SLC7A14

SLC7A13

SLC7A11

SLC7A10

SLC6A20

SLC6A19

SLC6A18

SLC6A17

SLC6A15

SLC6A14

SLC6A13

SLC6A12

SLC6A11

SLC66A1

SLC47A1

SLC46A1

SLC45A2

SLC44A1

SLC43A2

SLC43A1

SLC39A8

SLC38A9

SLC38A8

SLC38A7

SLC38A6

SLC38A5

SLC38A4

SLC38A3

SLC38A2

SLC38A1

SLC36A4

SLC36A3

SLC36A2

SLC36A1

SLC35D2

SLC35B3

SLC35B2

SLC32A1

SLC26A2

SLC26A1

SLC25A2

SLC25A1

SLC22A5

SLC22A4

SLC22A2

SLC19A3

SLC19A2

SLC19A1

SLC17A8

SLC17A7

SLC17A6

SLC17A5

SLC16A2

SLC15A4

SLC13A3

SLC12A2

SLC11A1

SLC10A7

SERINC5

SERINC4

SERINC3

SERINC2

SERINC1

SEPSECS

RPL36AL

RPL26L1

RPL22L1

NOXRED1

NDUFB10

NDUFAB1

NDUFA13

NAALAD2

MTHFD2L

MT-ATP6

MICOS13

METTL16

HSD17B8

HSD17B4

HMGCLL1

GLYATL1

GDAP1L1

GAL3ST4

GAL3ST3

CIAPIN1

CARNMT1

B4GALT6

B4GALT5

B4GALT4

B4GALT3

B4GALT2

B4GALT1

B3GALT6

ATP5F1A

ARL6IP5

ARL6IP1

ALDH9A1

ALDH8A1

ALDH7A1

ALDH6A1

ALDH5A1

ALDH4A1

ALDH1A1

AGTPBP1

ADORA2A

ABHD14B

UNC13B

TXNRD1

TMEM70

THNSL2

TALDO1

SUCLG2

SUCLG1

SUCLA2

STXBP1

STAT5B

STAT5A

SPOCK3

SPOCK2

SNCAIP

SNAP25

SLC7A9

SLC7A8

SLC7A7

SLC7A6

SLC7A5

SLC7A4

SLC7A3

SLC7A2

SLC7A1

SLC6A9

SLC6A8

SLC6A7

SLC6A6

SLC6A5

SLC6A1

SLC5A7

SLC5A6

SLC5A5

SLC3A2

SLC3A1

SLC1A7

SLC1A6

SLC1A5

SLC1A4

SLC1A3

SLC1A2

SLC1A1

SEPHS2

SEPHS1

RPS4Y2

RPS4Y1

RPS27L

RPS27A

RPS15A

RPL39L

RPL37A

RPL36A

RPL35A

RPL27A

RPL23A

RPL18A

RPL13A

RPL10L

RPL10A

RIMKLB

RIMKLA

PTGES2

PSPHP1

PSMD14

PSMD13

PSMD12

PSMD11

PSMD10

PSMB11

PSMB10

PRODH2

PPFIA4

PPFIA3

PPFIA2

PPFIA1

PNPLA8

PM20D1

PHYKPL

PET117

PDZD11

PAPSS2

PAPSS1

NMRAL1

NFKBIE

NFE2L2

MT-TL1

MT-ND6

MT-ND5

MT-ND4

MT-ND3

MT-ND2

MT-ND1

MTHFD1

MMADHC

MMACHC

MICAL2

MICAL1

LRRC8E

LRRC8D

LRRC8C

LRRC8A

LMBRD1

KCNJ10

HS3ST5

HMGCS2

HMGCS1

HIBADH

GUCY2D

GSTT2B

GOT1L1

GLYCTK

GGTLC3

GGTLC2

GGTLC1

FOLH1B

ENOSF1

ENOPH1

ELOVL7

ELOVL6

ELOVL5

ELOVL4

ELOVL3

ELOVL2

ELOVL1

EHHADH

EGFLAM

EEFSEC

EEF1E1

DHTKD1

DHFRP1

DGLUCY

DALRD3

CREBBP

COX6B1

CKMT1B

CKMT1A

CIAO2B

CIAO2A

CHST15

CHST14

CHST13

CHST12

CHST11

CARNS1

BOLA2B

BCKDHB

BCKDHA

B4GAT1

B3GNT7

B3GNT4

B3GNT3

B3GNT2

AVPR1A

ATP2B4

ATP1A2

ASRGL1

ASNSD1

ANGPT1

AMDHD1

AKR1A1

AHCYL2

AHCYL1

ADORA1

ADHFE1

ACSM2B

ACSM2A

ACSBG2

ACSBG1

ACOT13

ACOT12

ACOT11

ACADSB

AARSD1

YARS1

XYLT1

WARS2

WARS1

VARS2

VARS1

VAMP2

UROC1

UBA52

TYRP1

TTC37

TTC36

TSTD1

TSPO2

TRPC4

TPST2

TPST1

TMLHE

TKTL1

THTPA

THEM5

THEM4

THAP4

TGFB2

TARS3

TARS2

TARS1

STX1A

SIRT4

SHMT2

SHMT1

SFXN5

SFXN4

SFXN3

SFXN2

SFXN1

SARS2

SARS1

SARDH

RRM2B

RPS4X

RPS3A

RPS29

RPS28

RPS27

RPS26

RPS25

RPS24

RPS23

RPS21

RPS20

RPS19

RPS18

RPS17

RPS16

RPS15

RPS14

RPS13

RPS12

RPS11

RPS10

RPLP2

RPLP1

RPLP0

RPL7A

RPL41

RPL3L

RPL39

RPL38

RPL37

RPL36

RPL35

RPL34

RPL32

RPL31

RPL30

RPL29

RPL28

RPL27

RPL26

RPL24

RPL23

RPL22

RPL21

RPL19

RPL18

RPL17

RPL15

RPL14

RPL13

RPL12

RPL11

RPL10

RIMS1

RARS2

RARS1

RAB3A

QRSL1

QARS1

PYCR3

PYCR2

PYCR1

PXMP2

PSMF1

PSME4

PSME3

PSME2

PSME1

PSMD9

PSMD8

PSMD7

PSMD6

PSMD5

PSMD4

PSMD3

PSMD2

PSMD1

PSMC6

PSMC5

PSMC4

PSMC3

PSMC2

PSMC1

PSMB9

PSMB8

PSMB7

PSMB6

PSMB5

PSMB4

PSMB3

PSMB2

PSMB1

PSMA8

PSMA7

PSMA6

PSMA5

PSMA4

PSMA3

PSMA2

PSMA1

PSEN1

PSAT1

PRODH

PRELP

PRDX1

PRAF2

PPM1L

PPM1K

PLOD3

PLOD2

PLOD1

PIPOX

PHGDH

PGAP1

PDZK1

PDHA2

PDHA1

PCBD1

PARS2

PARK7

P4HA2

OTUB2

OPLAH

NUDT7

NUBPL

NUBP2

NUBP1

NTSR1

NTRK2

NR1H4

NPY5R

NDST4

NDST3

NDST2

NDST1

NDOR1

NAT8L

NARS2

NARS1

NADK2

NAA80

NAA60

NAA50

NAA40

NAA35

NAA30

NAA25

NAA20

NAA16

NAA15

NAA11

NAA10

MT-TW

MT-TV

MT-TK

MTHFS

MTHFR

MRPL3

MMS19

MLYCD

MIR21

MIPEP

MGST3

MGST2

MGST1

MECP2

MCCC2

MCCC1

MAT2B

MAT2A

MAT1A

MARS2

MARS1

LYRM4

LLGL2

LIPT2

LIPT1

LCMT1

LARS2

LARS1

KYAT3

KYAT1

KCTD7

KAT2B

KARS1

ITGB1

ISCA2

ISCA1

ILVBL

IL4I1

IBA57

IARS2

IARS1

HYAL4

HYAL1

HTR1B

HSPA9

HPRT1

HPGDS

HOGA1

HNF4A

HMGCL

HIBCH

HARS2

HARS1

HACD2

HACD1

GSTZ1

GSTT4

GSTT2

GSTT1

GSTP1

GSTO2

GSTO1

GSTM5

GSTM4

GSTM3

GSTM2

GSTM1

GSTK1

GSTA5

GSTA4

GSTA3

GSTA2

GSTA1

GRHPR

GPLD1

GPAT4

GPAA1

GLYAT

GLUD2

GLUD1

GLRX5

GLRX3

GLRX2

GIPC1

GGTA1

GGT3P

GFPT2

GFPT1

GDAP1

GARS1

GALNS

GADL1

G6PC2

FOLR3

FOLR2

FOLR1

FOLH1

FITM2

FBXL4

FARSB

FARSA

FARS2

ETNK1

ETHE1

ERCC5

ERCC4

ERCC3

ERCC2

EPRS1

EP300

ENPP1

EEF1G

ECHS1

EARS2

DUOX2

DUOX1

DPEP1

DMGDH

DIP2A

DHFR2

DGAT2

DGAT1

DDAH2

DDAH1

DARS2

DARS1

CTPS2

CTPS1

CSPG5

CSPG4

CPLX1

COX8A

COX5A

COX10

CNDP2

CLTRN

CLIC5

CLIC3

CLIC2

CLIC1

CKMT2

CIAO3

CIAO1

CHSY3

CHSY1

CHST9

CHST8

CHST7

CHST6

CHST5

CHST4

CHST3

CHST2

CHST1

CHPF2

CHAC2

CHAC1

CARS2

CARS1

BPNT2

BPNT1

BOLA2

BHMT2

BCKDK

BCAT2

BCAT1

BBOX1

AZIN2

AZIN1

ATP7A

ATG12

ATCAY

ASMTL

APBA1

AIMP2

AIMP1

AGXT2

AGMAT

AGBL4

AGBL1

AFMID

ADSS2

ADSS1

ADH1C

ACSS2

ACSS1

ACSM6

ACSM5

ACSM4

ACSM3

ACSM1

ACSL6

ACSL5

ACSL4

ACSL3

ACSL1

ACSF3

ACSF2

ACOT9

ACOT8

ACOT7

ACOT6

ACOT4

ACOT2

ACOT1

ACMSD

ACBD6

ACAT1

ACADM

ACAD8

ACACB

ACACA

ACAA1

ABCD4

ABCD1

ABCC5

ABCC2

ABCC1

AASDH

AARS2

AARS1

AANAT

AADAT

VCAN

UPB1

UGDH

TYMP

TXN2

TSHB

TPK1

TPH2

TPH1

TECR

TDO2

SYT1

SV2A

SUOX

SQOR

SOD1

SMOX

SEM1

SDSL

SDHA

SCO1

SCLY

SCD5

SAT1

RPSA

RPS9

RPS8

RPS7

RPS6

RPS5

RPS3

RPS2

RPL9

RPL8

RPL7

RPL6

RPL5

RPL4

RPL3

RIDA

RGS4

RGS2

QDPR

PSTK

PSPH

PRG3

PPT2

PPT1

PPCS

PPAT

PPA2

PPA1

POLG

PNPO

PNMT

PMVK

PLAU

PIGT

PIGS

PIGK

PFAS

PER2

PEPD

PEMT

PDPR

PDPN

PDP2

PDP1

PDK4

PDK3

PDK2

PDK1

PDHX

PDHB

PCK1

PCCB

PCCA

PAOX

P4HB

OXSM

OGDH

ODC1

OCA2

OAZ3

OAZ2

OAZ1

NQO1

NOX4

NOS3

NOS2

NOS1

NNMT

NIT2

NFU1

NFS1

NDNF

NCAN

NAT8

NAGS

MTRR

MTR

MTO1

MTAP

MSRA

MRM2

MRI1

MPST

MPC2

MMUT

MMAB

MMAA

MDH2

MDH1

MCEE

MAOA

LRP2

LIAS

LDHA

KYNU

KERA

ISCU

IRGM

IRF6

INMT

IDUA

IDO2

IDO1

IDH1

ICMT

HYKK

HTD2

HSCB

HRH3

HPDL

HNMT

HLCS

HEXB

HEXA

HAO1

HAGH

HADH

HAAO

GRM7

GRM2

GRM1

GPX1

GPT2

GPAM

GOT2

GOT1

GNMT

GMPS

GLUL

GLS2

GLO1

GLDC

GLCE

GLB1

GJA1

GGT7

GGT6

GGT5

GGT2

GGT1

GGCT

GCSH

GCLM

GCLC

GCH1

GCDH

GCAT

GATM

GATC

GATB

GART

GAMT

GAD2

GAD1

G6PD

FTCD

FPGS

FN3K

FMOD

FBP1

FASN

FAR2

FAR1

EXT2

EXT1

DSEL

DPYS

DPYD

DLST

DLAT

DIO3

DIO2

DIO1

DHPS

DHFR

CTNS

CSAD

CRYM

CPS1

COQ9

COLQ

CLN8

CLN3

CHPF

CHDH

CDO1

CBSL

CBR4

CA5A

BPHL

BLMH

BHMT

BCAN

BAAT

ATG7

ATG5

ATF4

ASS1

ASPG

ASPA

ASNS

ASMT

ART4

ARSG

ARSB

ARG2

ARG1

APIP

AOC3

AMD1

AHCY

AGXT

ADI1

ADH7

ADH5

ADH4

ACY1

ACP3

ACO2

ACLY

ACE2

ACCS

ACAN

ABAT

AASS

UST

TYR

TTL

TST

TRH

TPO

TDH

TAT

SRR

SRM

SPR

SP1

SMS

SDS

SCD

PTS

PKM

PAH

OTC

OMD

OGN

OAT

MVK

MVD

MTR

MPO

LUM

LPO

LEP

KMO

IYD

IVD

INS

IDS

HPD

HGD

HFE

HDC

HAL

GSS

GSR

GPT

GNS

GLS

GHR

FXN

FAU

FAH

ESD

DSE

DLD

DDO

DDC

DCT

DCN

DBT

DBI

DBH

DAO

CTH

CKM

CKB

CGA

CCK

CBS

CAD

BTD

BGN

AVP

AUH

ASL

APP

AMT

AGA

ADO

ADK

A2M

TH

PC

FH

CS
