# Supplementary material for: Glutamine Transporter SLC1A5 Regulates Ionizing Radiation-Derived Oxidative Damage and Ferroptosis
Source: Oxid Med Cell Longev. 2022 Oct 10;2022:3403009. doi: 10.1155/2022/3403009 (PMC9576409; doi:10.1155/2022/3403009)
Supplement: Supplementary Materials — Figure S1: the pie graph of targeted metabolomics. HepG2 cell line and HepG2-IRR cells were treated with Erastin (50 μM) for 6 hours, and cells were collected for targeted metabolomics. The content of amino acids (AA) accounting for 25% was the most abundant metabolites in HepG2-IRR cells (FDR-corrected p < 0.05; FC > 1.5), compared to wild-type HepG2 cells. Fatty acids account for 21% in HepG2-IRR cells after Erastin stimulation. Supplementary 1. The materials and protocol of targeted metabolomics. In brief, HepG2 cell line and HepG2-IRR cells were treated with Erastin (50 μM) for 6 hours, and cells were collected for targeted metabolomics. An ultraperformance liquid chromatography coupled to tandem mass spectrometry (UPLC-MS/MS) system was used to quantitate metabolites. Supplementary 2. The list of amino acid metabolism genes. Supplementary 3. The list of ferroptosis-related genes. Supplementary 4. Twenty amino acid-ferroptosis genes were identified as prognostic factors for predicting the prognosis of liver tumor patients. Supplementary 5. SLC1A5, SLC7A11, TXNRD1, and ASNS are the independent prognostic factors for liver tumor patients. [file 3403009.f1.zip › supplementary 1.pdf]

❖ Materials and Methods

1. Chemicals and Reagents

All of the standards were obtained from Sigma-Aldrich (St. Louis, MO, USA), Steraloids Inc. (Newport, RI, USA) and TRC Chemicals (Toronto, ON, Canada). All the standards were accurately weighed and prepared in water，methanol, sodium hydroxide solution, or hydrochloric acid solution to obtain individual stock solution at a concentration of 5.0 mg/mL. Appropriate amount of each stock solution was mixed to create stock calibration solutions.

Formic acid was of Optima grade and obtained from Sigma-Aldrich (St. Louis, MO, USA). Methanol (Optima LC-MS), acetonitrile (Optima LC-MS), and isopropanol (Optima LC-MS) were purchased from Thermo-Fisher Scientific (FairLawn, NJ, USA). Ultrapure water was produced by a Mill-Q Reference system equipped with a LC-MS Pak filter (Millipore, Billerica, MA, USA).

2. Sample Preparation

Each cell sample that was harvested and stored in an Eppendorf Safelock microcentrifuge tube, was mixed with 10 pre-chilled zirconium oxide beads and 20μL of deionized water. The sample was homogenated for 3 minutes and 150μL of Methanol containing internal standard was added to extract the metabolites. The sample was homogenated for another 3 minutes and then centrifuged at 18000g for 20 minutes. Then the supernatant was transferred to a 96-well plate. The following procedures were performed on a Eppendorf epMotion Workstation(Eppendorf Inc., Humburg, Germany). 20μL of freshly prepared derivative reagents was added to each well. The plate was sealed and the derivatization was carried out at 30° Cfor 60 min. After derivatization, the sample was evaporated for 2h. 330μL of ice-cold 50% methanol solution was added to reconstitute the sample. Then the plate was stored at -20° Cfor 20 minutes and followed by 4000g centrifugation at 4 °C for 30 minutes. 135μL of supernatant was transferred to a new 96-well plate with 10μL

internal standards in each well. Serial dilutions of derivatized stock standards were added to the left wells.　Finally the plate was sealed for LC-MS analysis.

3. Instrumentation

A ultra-performance liquid chromatography coupled to tandem mass spectrometry (UPLC-MS/MS) system (ACQUITY UPLC-Xevo TQ-S, Waters Corp., Milford, MA, USA) was used to quantitate a l l t a r g e t e d metabolite s in this project.The optimized instrument settings are briefly described below. The instrument performance optimization and routine maintenance were performed every week.

UPLC-MS/MS instrument settings	
**UPLC**	
**Column**	ACQUITY UPLC BEH C18 1.7 µM VanGuard pre-column (2.1×5 mm) and ACQUITY UPLC BEH C18 1.7 µM analytical column (2.1 × 100 mm)
**Column Temp. (°C)**	40
**Sample Manager Temp. (°C)**	10
**Mobile Phases**	A=water with 0.1% formic acid; and B=acetonitrile / IPA (70:30)
**Gradient Conditions**	0-1 min (5% B), 1-11min (5-78% B), 11-13.5 min (78-95% B), 13.5-14 min (95-100% B), 14-16 min (100% B)，16-16.1 min (100-5% B)，16．1-18 min (5% B).
**Flow Rate (mL/min)**	0.40
**Injection Vol. (µl)**	5.0
**MASS SPECTROMETER**	
**Capillary (Kv)**	1.5 (ESI+), 2.0 (ESI-)
**Source Temp (°C)**	150
**Desolvation Temp (°C)**	550
**Desolvation Gas Flow (L/Hr)**	1000

4. Analytical Quality Control Procedures

The rapid turnover of many intracellular metabolites makes immediate metabolism quenching necessary. The extraction solvents are stored in -20°C freezer overnight and added to the samples immediately after the samples were thawed. We use ice-salt bath to keep the samples at a low temperature and minimize sample degradation during sample preparation. All the prepared samples should be analyzed within 48 hours after sample extraction and derivatization.

A comprehensive set of rigorous quality control/assurance procedures is employed to ensure a consistently high quality of analytical results, throughout controlling every single step from sample receipt at laboratory to final deliverables. The ultimate goal of QA/QC is to provide the reliable data for biomarker discovery study and/ or to aid molecular biology research. To achieve this, three types of quality control samples i.e., test mixtures, internal standards, and pooled biological samples are routinely used in our metabolomics platform. In addition to the quality controls, conditioning samples, and solvent blank samples are also required for obtaining optimal instrument performance.

Test mixtures comprise a group of commercially available standards with a mass range across the system mass range used for the study samples. These samples were analyzed at the beginning and end of each batch run to ensure that the instruments were performing within laboratory specifications (retention time stability, chromatographic peak shape, and peak signal intensity). The retention time shift should be within 4 sec. and the difference of peak intensity should be within 15% for LC-MS.

Internal standards were added to the test samples in order to monitor analytical variations during the entire sample preparation and analysis processes. The Pooled QC samples were prepared by mixing aliquots of the study samples such that the pooled samples broadly represent the biological average of the whole sample set. The

QC samples for this project were prepared with the test samples and injected at regular intervals (after every 14 test samples for LC-MS) throughout the analytical run.

Reagent blank samples are a mixture of solvents used for sample preparation and are commonly processed using the same procedures as the samples to be analyzed. The reagent blanks serve as a useful alert to systematic contamination. As the reagent blanks consist of high purity solvents and are analyzed using the same methods as the study samples, they are also used to wash the column and remove cumulative matrix effects throughout the study.

The calibrators consist of a blank sample (matrix sample processed without internal standard), a zero sample (matrix sample processed with internal standard), and a series of seven concentrations covering the expected range for the metabolites present in the specific biological samples. LLOQ and ULOQ are the lowest and highest concentration of the standard curve that can be measured with acceptable accuracy and precision.

5. Sample Run Order

To diminish analytical bias within the entire analytical process, the samples were analyzed in group pairs but the groups were analyzed randomly. The QC samples, calibrators, and blank samples were analyzed across the entire sample set.

6. Sample Control Procedure (ISO9001, QAIC/CN/170149)

Each sample received was accessioned into Metabo-Profile LIMS system and was assigned by the LIMS a unique identifier, which was associated with the original source identifier only. This identifier was used to track all sample handling, tasks, results etc. The samples and aliquots were bar-coded and tracked by the LIMS system. All portions of any sample were automatically assigned their own unique identifiers by the LIMS when a new task was created; the relationship of these samples was also

tracked. All samples were maintained at -80 °C until processed. Metabo -Profile will help customers store the remaining samples for up to six months before return or disposal. A formal disposal form authorized by the customer should be acquired.

7. Data Control Procedure (ISO9001, QAIC/CN/170149)

The data retained on instrument control computers are immediately removed and transferred to a local data server (Metabo-Profile) for further data analysis located in a locked room. Metabo-Profile will not share any information with other customers until permitted. The data will be retained on Metabo-Profile server for up to 6 months.

8. Data Analysis

Software

The raw data files generated by UPLC-MS/MS were processed using the MassLynx software (v4.1, Waters, Milford, MA, USA) to perform peak integration, calibration, and quantitation for each metabolite. The self-developed platform **iMAP**（v1.0, Metabo-Profile, Shanghai, China）was used for statistical analyses, including PCA, OPLS-DA, univariate analysis and pathway analysis, et al.

Quantitation

Mass spectrometry-based quantitative metabolomics refers to the determination of the concentration of a substance in an unknown sample by comparing the unknown to a set of standard samples of known concentration (i.e., calibration curve). The calibration curve is a plot of how the analytical signal changes with the concentration of the analyte (the substance to be measured). For most analyses a plot of instrument response vs. concentration will show a linear relationship. This yields a model described by the equation $y = ax + b$, where y is the instrument response e.g., peak height or area, a represents the slope/sensitivity, and b is a constant that describes the background. The analyte concentration (x) of unknown samples may be calculated from this equation.

Statistics

Our proprietary software can perform a collection of data processing, interpretation, and visualization. For many metabolomics studies, two types of statistical analysis are extensively performed: 1) multivariate statistical analyses such as principal component analysis (PCA), partial least square discriminant analysis (PLS-DA), orthogonal partial least square discriminant analysis (OPLS-DA), random forest, support vector machine learning and so on; 2) univariate statistical analyses including student t-test, Mann-Whitney-Wilcoxon (U-test), ANOVA, correlation analysis, etc. The optimal choice of statistical methods is often driven by the data and the project goals.

Multivariate Statistical Analyses

Principal Components Analysis (PCA) is an unsupervised modeling method commonly used to detect data outliers, clustering, and classification trends without a priori knowledge of the sample set. The principal components (PCs) that are derived from the data set are orthogonal to one another and reflect reducing levels of the variation in the data set. Thus, the first principal component (PC1) expresses more variation than the second principal component (PC2), which, in turn, expresses more variation than PC3, and so on. In this way, PCA significantly decreases the number of variables (scores) required for representation of the data set and allows for visualization of data clusters and outliers based on multiple variables in two- or three-dimensional space. Since metabolomics data usually contain very complex biological information such as dietary intervention, lifestyle, aging, gender, region-dependent environmental factors, and diseases, it is extremely difficult to discriminate the effect of one individual factor from the broader biological information. Therefore, sophisticated multivariate statistical modeling such as partial least square discriminant analysis (PLS-DA) and/or orthogonal partial least square discriminant analysis (OPLS-DA) has been extensively used for multi-class classification and identification of differently altered metabolites.

Partial Least Square Discriminant Analysis (PLS-DA) is a generalized multiple regression method that can deal with multiple collinear X (herein mass

spectral data) and Y (classes) variables. This method has two objectives: one is to approximate X and Y, and the other is to model the relationship between them. Since this method is able to maximize the separation between classes using a pre-defined approach, it is widely-used in the metabolomics field to handle the complex data sets generated from a complicated biological matrix. In the current project, PLS-DA modeling is used as a multi-class classifier to visualize the difference between global metabolic profiles among the given groups that provides more valuable information beyond what can be gleaned from PCA.

The OPLS method is a recent modification of the PLS method. The **Orthogonal Partial Least Square Discriminant Analysis (OPLS-DA)** algorithm decomposes the raw data set into three parts: systemic variations, orthogonal/unrelated information, and residual. This leads to a model with a minimal number of predictive components defined by the number of degrees of freedom (k − 1 dimensions) between group variances (k classes). This partitioning of the X-data facilitates model interpretation and model prediction.

Interpretation of models. In PCA, PLS-DA, or OPLS-DA, each spatial dot in the K-dimensional space represents an individual sample with the samples color-coded according to grouping information. R2X and R2Y represent the fraction of the variance of X matrix and Y matrix, respectively, while Q2Y suggests the predictive accuracy of the model. Cumulative values of R2X, R2Y and Q2Y close to 1.0 indicate an excellent model with a reliable predictive ability. Those variables with variable influence on projection (VIP) greater than 1.0 are considered significantly different between classes.

Data quality control and validation of modeling. To validate the model against over-fitting, a 7-round cross-validation was carried out with 1/7th of the sample set being excluded from the modeling in each round. Using this method, the Y value for each subject was predicted using a model from which that subject has been excluded during the model building and all the predictions are collated. The resulting R2Y provides an estimate of how well the model fits the Y data (herein classification information) and Q2Y provides an estimate of how well the model predicts the Y data.

The cumulative values of R2Ycum and Q2Ycum approaching 1.0 indicate a reliable model with a satisfactory predictive ability.

References

1. Guoxiang Xie, Lu Wang, Tianlu Chen, et al. A Metabolite Array Technology for Precision Medicine. Anal. Chem. 2021, 93, 14, 5709–5717.
