# Supplementary material for: Glutamine Transporter SLC1A5 Regulates Ionizing Radiation-Derived Oxidative Damage and Ferroptosis
Source: Oxid Med Cell Longev. 2022 Oct 10;2022:3403009. doi: 10.1155/2022/3403009 (PMC9576409; doi:10.1155/2022/3403009)
Supplement: Supplementary Materials — Figure S1: the pie graph of targeted metabolomics. HepG2 cell line and HepG2-IRR cells were treated with Erastin (50 μM) for 6 hours, and cells were collected for targeted metabolomics. The content of amino acids (AA) accounting for 25% was the most abundant metabolites in HepG2-IRR cells (FDR-corrected p < 0.05; FC > 1.5), compared to wild-type HepG2 cells. Fatty acids account for 21% in HepG2-IRR cells after Erastin stimulation. Supplementary 1. The materials and protocol of targeted metabolomics. In brief, HepG2 cell line and HepG2-IRR cells were treated with Erastin (50 μM) for 6 hours, and cells were collected for targeted metabolomics. An ultraperformance liquid chromatography coupled to tandem mass spectrometry (UPLC-MS/MS) system was used to quantitate metabolites. Supplementary 2. The list of amino acid metabolism genes. Supplementary 3. The list of ferroptosis-related genes. Supplementary 4. Twenty amino acid-ferroptosis genes were identified as prognostic factors for predicting the prognosis of liver tumor patients. Supplementary 5. SLC1A5, SLC7A11, TXNRD1, and ASNS are the independent prognostic factors for liver tumor patients. [file 3403009.f1.zip › supplementary 3.docx]

RPL8

IREB2

ATP5MC3

CS

EMC2

ACSF2

NOX1

CYBB

NOX3

NOX4

NOX5

DUOX1

DUOX2

G6PD

PGD

VDAC2

PIK3CA

FLT3

SCP2

TP53

ACSL4

LPCAT3

NRAS

KRAS

HRAS

TF

TFRC

TFR2

SLC38A1

SLC1A5

GLS2

GOT1

CARS1

ALOX5

KEAP1

HMOX1

ATG5

ATG7

NCOA4

ALOX12

ALOX12B

ALOX15

ALOX15B

ALOXE3

PHKG2

ACO1

G6PDX

ULK1

ATG3

ATG4D

BECN1

MAP1LC3A

GABARAPL2

GABARAPL1

ATG16L1

WIPI1

WIPI2

SNX4

ATG13

ULK2

SAT1

EGFR

MAPK3

MAPK1

BID

ZEB1

DPP4

CDKN2A

PEBP1

SOCS1

CDO1

MYB

MAPK8

MAPK9

CHAC1

MAPK14

LINC00472

PRKAA2

PRKAA1

ELAVL1

BAP1

ABCC1

MIR6852

ACVR1B

TGFBR1

EPAS1

HILPDA

HIF1A

IFNG

ANO6

LPIN1

HMGB1

TNFAIP3

TLR4

ATF3

ATM

YY1AP1

EGLN2

MIOX

TAZ

MTDH

IDH1

SIRT1

FBXW7

PANX1

DNAJB6

BACH1

LONP1

CD82

IL1B

CTSB

POR

CYB5R1

ELOVL5

FADS1

FBW7

PTEN

NR1D1

NR1D2

TBK1

IL6

USP7

miR-182-5p

miR-378a-3p

ATF4

AQP3

AQP5

AQP8

LINC00618

MT1DP

PEX10

AGPAT3

PEX12

CHP1

GPAT4

BRPF1

OSBPL9

INTS2

MMD

CYP4F8

MLLT1

TTPA

GRIA3

EPT1

POM121L12

LIG3

AEBP2

AGPS

CDCA3

PEX2

PEX6

TIMM9

DCAF7

LCE2C

FAR1

PHF21A

SMAD7

LYRM1

AMN

PEX3

MTCH1

ACADSB

PVT1

hsa_circ_0008367

SLC39A14

MAP3K11

GSK3B

PTGS2

DUSP1

NOS2

NCF2

MT3

UBC

ALB

TXNRD1

SRXN1

GPX2

BNIP3

OXSR1

SELENOS

ANGPTL7

SLC7A11

DDIT4

LOC284561

ASNS

TSC22D3

DDIT3

JDP2

SESN2

SLC1A4

PCK2

TXNIP

VLDLR

GPT2

PSAT1

LURAP1L

SLC7A5

HERPUD1

XBP1

SLC3A2

CBS

ZNF419

KLHL24

TRIB3

ZFP69B

ATP6V1G2

VEGFA

GDF15

TUBE1

ARRDC3

CEBPG

SNORA16A

RGS4

BLOC1S5-TXNDC5

LOC390705

EIF2S1

KIM-1

CXCL2

RELA

HSD17B11

SETD1B

FTL

MAFG

IL33

FTH1

SLC40A1

GPX4

HAMP

HSPB1

NFE2L2

STEAP3

DRD5

DRD4

MAP3K5

SLC2A1

SLC2A3

SLC2A6

SLC2A8

SLC2A12

GLUT13

SLC2A14

EIF2AK4

TFAP2C

SP1

HBA1

NNMT

PLIN4

HIC1

STMN1

RRM2

CAPG

HNF4A

NGB

YWHAE

GABPB1

AURKA

MIR4715

RIPK1

PRDX1

MIR30B

MMP13

LRRFIP1

AKR1C1

AKR1C2

AKR1C3

RB1

HSF1

GCLC

SQSTM1

NQO1

MUC1

MT1G

CISD1

FANCD2

FTMT

HSPA5

HELLS

SCD

FADS2

SRC

STAT3

PML

MTOR

NFS1

TP63

CDKN1A

MIR137

ENPP2

FH

CISD2

MIR9-1

MIR9-2

MIR9-3

ISCU

ACSL3

OTUB1

CD44

LINC00336

BRD4

PRDX6

MIR17

NF2

ARNTL

JUN

CA9

TMBIM4

PLIN2

MIR212

Fer1HCH

AIFM2

LAMP2

ZFP36

PROM2

CHMP5

CHMP6

CAV1

GCH1

SIRT3

DAZAP1

PIR

HCAR1

SLC16A1

NR4A1

RPTOR

SREBF1

SREBF2

FZD7

P4HB

NT5DC2

BCAT2

PLA2G6

MIR424

PARK7

FXN

SUV39H1

ATF2

ACOT1

ALDH3A2

STK11

FNDC5

CircIL4R

CDH1

MIR214

NEDD4L

BRD2

BRD3

BRDT

DECR1
