# Supplementary material for: Glutamine Transporter SLC1A5 Regulates Ionizing Radiation-Derived Oxidative Damage and Ferroptosis
Source: Oxid Med Cell Longev. 2022 Oct 10;2022:3403009. doi: 10.1155/2022/3403009 (PMC9576409; doi:10.1155/2022/3403009)
Supplement: Supplementary Materials — Figure S1: the pie graph of targeted metabolomics. HepG2 cell line and HepG2-IRR cells were treated with Erastin (50 μM) for 6 hours, and cells were collected for targeted metabolomics. The content of amino acids (AA) accounting for 25% was the most abundant metabolites in HepG2-IRR cells (FDR-corrected p < 0.05; FC > 1.5), compared to wild-type HepG2 cells. Fatty acids account for 21% in HepG2-IRR cells after Erastin stimulation. Supplementary 1. The materials and protocol of targeted metabolomics. In brief, HepG2 cell line and HepG2-IRR cells were treated with Erastin (50 μM) for 6 hours, and cells were collected for targeted metabolomics. An ultraperformance liquid chromatography coupled to tandem mass spectrometry (UPLC-MS/MS) system was used to quantitate metabolites. Supplementary 2. The list of amino acid metabolism genes. Supplementary 3. The list of ferroptosis-related genes. Supplementary 4. Twenty amino acid-ferroptosis genes were identified as prognostic factors for predicting the prognosis of liver tumor patients. Supplementary 5. SLC1A5, SLC7A11, TXNRD1, and ASNS are the independent prognostic factors for liver tumor patients. [file 3403009.f1.zip › supplementary 4.docx]

| gene | conMean | treatMean | logFC | pValue | fdr |
| --- | --- | --- | --- | --- | --- |
| SLC38A1 | 1.293731 | 4.376645 | 1.758287 | 3.00E-05 | 4.18E-05 |
| NOS2 | 0.097262 | 0.402505 | 2.049054 | 5.21E-13 | 1.53E-12 |
| SLC1A4 | 1.912718 | 5.006494 | 1.388176 | 6.03E-15 | 2.00E-14 |
| TXNRD1 | 6.763141 | 28.10662 | 2.055145 | 1.93E-17 | 8.54E-17 |
| ASNS | 0.500574 | 2.570302 | 2.360284 | 2.43E-09 | 5.14E-09 |
| NNMT | 469.383 | 140.6666 | -1.73849 | 1.15E-17 | 6.09E-17 |
| G6PD | 1.311715 | 13.58215 | 3.372185 | 6.03E-25 | 1.60E-23 |
| BCAT2 | 3.151893 | 8.319695 | 1.400312 | 2.26E-18 | 1.33E-17 |
| SLC1A5 | 2.729119 | 8.796147 | 1.688436 | 0.004123 | 0.004966 |
| SLC7A11 | 0.030108 | 0.889909 | 4.885429 | 1.65E-19 | 1.25E-18 |
| ACSL4 | 7.32149 | 46.12191 | 2.655243 | 3.57E-12 | 8.61E-12 |
| ABCC1 | 0.707154 | 2.380374 | 1.751092 | 3.82E-06 | 5.63E-06 |
| NOX4 | 0.021637 | 0.24261 | 3.487096 | 1.94E-28 | 1.03E-26 |
| SLC7A5 | 2.462716 | 5.037566 | 1.032477 | 0.026349 | 0.030359 |
| DUOX1 | 0.056932 | 0.494106 | 3.117501 | 5.88E-20 | 5.20E-19 |
| GLS2 | 4.778707 | 1.786804 | -1.41924 | 1.24E-15 | 4.40E-15 |
| NQO1 | 1.459665 | 57.95383 | 5.311194 | 6.82E-16 | 2.78E-15 |
| CS | 5.378785 | 13.81835 | 1.361233 | 1.45E-22 | 2.51E-21 |
| ACADSB | 72.1887 | 32.77565 | -1.13915 | 1.46E-18 | 9.69E-18 |
| RPL8 | 211.6811 | 704.8365 | 1.735396 | 4.54E-21 | 4.82E-20 |
